# Who Will Continuously Depend on Compression to Control Persistent or Progressive Breast Cancer-Related Lymphedema Despite 2 Years of Conservative Care?

**DOI:** 10.3390/jcm9113640

**Published:** 2020-11-12

**Authors:** Chul Jung, JaYoung Kim, Yu Jin Seo, Kyeong Joo Song, Ma. Nessa Gelvosa, Jin Geun Kwon, Changsik John Pak, Hyunsuk Peter Suh, Joon Pio Hong, Hwa Jung Kim, Jae Yong Jeon

**Affiliations:** 1 Department of Rehabilitation Medicine, Asan Medical Center, University of Ulsan College of Medicine, 88 Olympic-ro 43-gil, Songpa-gu, Seoul 05505, Korea; speciron90@gmail.com (C.J.); iris_jy@naver.com (J.K.); ujingirl@naver.com (Y.J.S.); song135@naver.com (K.J.S.); nicegelvosa@gmail.com (M.N.G.); 2 Department of Plastic and Reconstructive Surgery, Asan Medical Center, University of Ulsan College of Medicine, Seoul 05505, Korea; ione2bbest@naver.com (J.G.K.); iloveps@amc.seoul.kr (C.J.P.); hpetersuh@gmail.com (H.P.S.); joonphong@amc.seoul.kr (J.P.H.); 3 Department of Clinical Epidemiology and Biostatics, Asan Medical Center, University of Ulsan College of Medicine, Seoul 05505, Korea; hello.hello.hj@gmail.com

**Keywords:** breast cancer, lymphedema, compression, predictor, physiologic surgery

## Abstract

Background: When a patient with breast cancer-related lymphedema (BCRL) depends on continuous compression management, that is, when interstitial fluid accumulation is continuously ongoing, surgical treatment should be considered. Physiologic surgery is considered more effective for early-stage lymphedema. The purpose of this study was to identify predictors of patients with BCRL who will be compression-dependent despite 2 years of conservative care. Methods: This study included patients with BCRL who followed up for 2 years. Patients were classified into two groups (compression-dependent vs. compression-free). We identified the proportion of compression-dependent patients and predictors of compression dependence. Results: Among 208 patients, 125 (60.1%) were classified into the compression-dependent group. Compression dependence was higher in patients with direct radiotherapy to the lymph nodes (LNs), those with five or more LNs resections, and those with BCRL occurring at least 1 year after surgery. Conclusions: BCRL patients with direct radiotherapy to the LNs, extensive LN dissection, and delayed onset may be compression-dependent despite 2 years of conservative care. Initially moderate to severe BCRL and a history of cellulitis also seem to be strongly associated with compression dependence. Our results allow for the early prediction of compression-dependent patients who should be considered for physiologic surgery.

## 1. Introduction

Lymphedema is a chronic and debilitating condition characterized by excess protein-rich fluid accumulation in interstitial tissues due to impairment of the lymphatic system. In developed countries, a secondary condition, known as breast cancer-related lymphedema (BCRL), is the most common form. Breast cancer treatment, such as lymph node dissection and radiotherapy, damages regional lymphatic circulation and can lead to BCRL, which presents as breast and arm swelling. Previous studies have reported that up to 40% of patients with breast cancer develop lymphedema [1,2,3,4].

The time course of BCRL can be divided into two categories, transient and continuous. Some patients with BCRL present with transient swelling, which may resolve spontaneously or with conservative management. Further surgical treatment for this group is not necessary. However, other patients have chronic lymphedema despite active conservative care [1,2,5]. A few previous reports have addressed this different time course and referred to it as temporary and persistent lymphedema [6,7,8]. Several other studies have reported this different course from the viewpoint of whether lymphedema progressed [9,10,11]. Between 40% and 60% of BCRLs are prone to be persistent [6,7,8] or progressive [9,10,11].

The therapeutic approach to BCRL varies on the time course that it presented with. BCRL management can be classified into conservative and surgical treatment. Currently, conservative management with complex decongestive therapy (CDT) is the standard care. CDT is mainly composed of manual lymphatic drainage (MLD), compression therapy, remedial exercise, and skin care and consists of several hospital-based sessions followed by home-based management [12,13]. Compression is the key to home-based programs, and it can be applied using bandages, stockings, or garments to decrease the retained interstitial fluid. CDT may be sufficient for temporary lymphedema, but surgical treatment should also be considered for persistent or progressive lymphedema [4,14,15,16].

In BCRL where interstitial fluid accumulation continues despite compression, surgical treatment should be considered for several reasons. First, lymphedema is associated with a series of pathologic changes in lymphatics. In brief, increased lymphatic pressure results in fibrosclerotic changes to the lymphatic vessels, and lymphatic stasis leads to chronic inflammation, causing progressive fat deposition in soft tissue. The sclerotic lymphatic vessels lose functions such as lymph transport [14,15,17,18]. In addition, long-term compression management burdens the patient and often diminishes the patient’s quality of life [19]. It has been suggested that further surgical treatment lowers the burden on patients and improves their quality of life by allowing compression to be reduced or discontinued [15,16,20,21,22,23].

Surgical treatment of lymphedema can be categorized into physiologic or debulking procedures. With recent advances in microsurgery, physiologic procedures, such as lymphaticovenous anastomosis (LVA) and vascularized lymph node transfer (VLNT), are the first choice for patients in need of surgical treatment. Physiologic surgery restores impaired lymphatic drainage by utilizing the remaining lymphatic and venous systems. Due to insufficient evidence available in literature, timing of the procedure typically depends on previous reports or the operator’s preference. However, due to the pathologic process and the possibility of decreased function in the residual lymphatics, it is commonly agreed that physiologic surgery may be more effective when performed earlier [15,16,18,22,23].

Moreover, the early period after lymphatic impairment is also critical for its restoration. Regeneration of the residual lymphatics has been reported to take place in patients with breast cancer who underwent an axillary dissection [24]. Although lymphatic regeneration is still under investigation, this might be an underlying mechanism for the effect of early intervention in patients with BCRL. Previous studies suggested that early intervention for BCRL can help prevent its progression and change its time course [4,25,26]. In one study, it was reported that patients who were subclinically diagnosed with BCRL by bioimpedance analysis could also benefit from conservative care, which led to resolution of their BCRL [26]. Taken together, all the patients with early-stage BCRL should be actively managed with conservative care; however, some of them whose lymphedema will be persistent should be considered for physiologic surgery.

Therefore, early prediction of patients who will present with persistent or progressive lymphedema despite active conservative care will decrease unnecessary surgery and improve the effectiveness of physiologic surgery. A few previous studies have reported predictive or risk factors for persistent [5,6,7,8] or progressive [9,10,11] lymphedema. However, these reports had the major limitation that they simply defined persistent or progressive lymphedema as increased limb volume without considering whether excess accumulation of the interstitial fluid was ongoing. The increased limb volume did not reflect real-time interstitial fluid accumulation because it was affected by pathologic changes such as fat deposition. In addition, these reports did not sufficiently address conservative care of patients.

The aim of the present study was to identify the predictors of BCRL patients with ongoing interstitial fluid accumulation, that is, patients who will remain dependent on compression management despite 2 years of conservative care.

## 2. Materials and Methods

### 2.1. Patients and Setting

We performed a retrospective, single-center cohort study. The study population included women over 18 years who underwent primary surgery for breast cancer in our institution and were referred to the outpatient lymphedema clinic. Women eligible for this study were diagnosed with BCRL and began management with CDT between August 2015 and December 2017. Only patients who were followed up for at least 2 years were included. We set 2 years for minimum follow-up period based on previous studies reporting the time course of lymphedema [1,5,8]. Patients were excluded if they had bilateral or stage IV breast cancer, underwent additional surgery to control their cancer or had severe cardiac or renal diseases causing generalized edema. After exclusion, the remaining patients were included in the final cohort. The protocol was reviewed and approved by the ethics committee of our institution (IRB No. 2020-0513).

### 2.2. Measurement and Diagnosis of BCRL

After primary breast cancer surgery, most patients were referred to the lymphedema clinic for BCRL screening. Patients were diagnosed with BCRL if they complained of subjective symptoms or exhibited objective findings of lymphedema over three consecutive follow-ups, corresponding to at least 3 to 6 months. Complaints of heaviness, tightness, or tenderness were considered as significant subjective symptoms. The objective findings mainly included increased subcutaneous thickness, pitting, and an increase in the circumferential difference between both arms. The physiatrist routinely measured the circumference at 10 cm distal and 5 cm and 10 cm proximal to the lateral epicondyle. If necessary, the circumference of wrist or a specifically swollen area was also measured. The follow-up interval varied from 1 to 3 months based on each patient’s clinical evaluation. In addition, bioimpedance analysis (BIA) and lymphoscintigraphy were performed as objective tests to confirm the diagnosis of BCRL. For BIA, the extracellular fluid (ECF) ratio (affected:unaffected side), the single frequency bioimpedance (SFBIA) ratio at 5 kHz (unaffected:affected side), and extracellular water volume (ECW) divided by total body water volume (TBW) were analyzed. Previously established cut-off values for diagnosis of lymphedema were used: ECF ratio ≥ 1.010, SFBIA ratio at 5 kHz ≥1.070/1.030 for at-risk dominant/non-dominant arms, and ECW/TBW ≥ 0.390 [27,28].

### 2.3. Treatment of Breast Cancer and BCRL

In our institution, multimodal breast cancer treatment was decided by a multidisciplinary approach and updated guidelines. At the lymphedema clinic, the physiatrist focused on surveillance and early intervention for BCRL.

Management of BCRL was based on the published guideline [29]. When a patient was diagnosed with BCRL, the physiatrist prescribed CDT and provided education on lymphedema management. The CDT program in our institution consisted of 5– or 10–one-hour sessions for 1 or 2 weeks, mainly focusing on MLD and compression therapy. After the hospital-based program, the patient was transited to home-based management and maintained with BCRL care [12,13]. Initial home-based management involved daily daytime wearing of elastic stockings and nighttime application of short-stretch bandages. At every follow-up, the physiatrist evaluated the lymphedema status and modified the frequency, duration, and device for compression management accordingly. Clinical decisions were aimed at tapering compression as much as possible while maintaining stable lymphedema status. If the patient maintained stable lymphedema status, such that the circumference of the swollen arm is decreasing or sustained or subjective symptoms improved, the physiatrist tried to decrease the frequency and duration of compression, and to replace bandages with stockings or garments. The frequency of compression was decreased to an interval of 2 or 3 days. Conversely, compression was increased if the patient exhibited increased arm circumference or experienced aggravated symptoms. Compliance to conservative care was also assessed by the physiatrist. Compliance was considered poor when the patient did not follow compression as advised for more than 2 follow-up visits.

### 2.4. Data Collection

Data were retrospectively collected via electronic medical records. Collected data were categorized based on patient demographics and tumor-, treatment-, and swelling-related characteristics. For swelling-related characteristics, the percentage of excess circumference (PEC) at the time of diagnosis of BCRL was used to express the severity of initial lymphedema. PEC was a modification of the percentage of excess volume, which had been used in previous studies [6,10,18,25]. PEC was defined as excess circumference, i.e., circumference difference between the lymphedematous arm (C_L_) and healthy arm (C_H_)), relative to the circumference of the healthy arm (C_H_) and calculated as PEC = ((C_L_ − C_H_)/C_H_) × 100%. The maximum PEC values measured at different sites on the patients’ arms were used in the study.

We also defined dependence on compression management as a clinical need for compression to control the lymphedema status. As previously described, the physiatrist tried tapering each patient’s compression to the minimum. Therefore, dependence on compression despite tapering efforts represented ongoing interstitial fluid accumulation due to an impaired lymphatic system. Based on whether patients with BCRL were compression-dependent at their 2-year follow-up, we classified them into two groups (compression-dependent vs. compression-free). Patients in the compression-dependent group required at least intermittent compression on a regular basis, whereas those in the compression-free group could control their BCRL status without regular compression management.

### 2.5. Outcomes

The primary outcome was determination of predictors associated with patients in the compression-dependent group, and the secondary outcomes were the proportion and clinical characteristics of patients in the compression-dependent group. The incidence and risk of a patient being categorized as compression-dependent based on the number of identified predictors were also investigated.

### 2.6. Statistical Analysis

All statistical analyses were conducted using PASW Statistics 18 (SPSS Inc., Chicago, IL, USA). A *p*-value < 0.05 was considered statistically significant. Continuous variables were presented as mean ± standard deviation, and categorical variables were presented as percentage. The Student’s *t*-test, Pearson’s χ^2^ test, and Fisher’s exact test were used to compare variables between the compression-dependent and compression-free groups.

The association between dependence on compression management and clinical variables was assessed using univariate logistic regression analysis. Multivariate logistic regression analysis with stepwise fashioned variable selection was used to identify predictors of a patient being categorized in the compression-dependent group. Variables with *p* < 0.1 in the univariate analysis were considered as possible predictors. All possible predictors, except tumor-related characteristics, were included in the multivariate analysis. Using univariate logistic regression analysis, the risk of a patient being compression-dependent was also evaluated based on the number of identified predictors.

## 3. Results

### 3.1. Study Population and Baseline Characteristics

A total of 486 patients who had been diagnosed with BCRL and started CDT between August 2015 and December 2017 were eligible for this study. Of these, 230 patients with follow-up periods of <2 years were excluded, and 256 patients were finally included in the study population. Patients with bilateral (*n* = 19) or stage IV (*n* = 18) breast cancer, those who had undergone additional surgery to control breast cancer (*n* = 10), and those with generalized edema due to heart failure (*n* = 1) were excluded. As a result, this cohort included 208 patients with BCRL. Of them, four presented with severely uncontrolled lymphedema despite maximal conservative care and underwent physiologic surgery during the follow-up period. Three of these four patients underwent LVA, while the other one underwent VLNT. These patients were classified into the compression-dependent group.

A summary of clinical characteristics is presented in Table 1. The mean age of 208 patients was 49.0 years, and the mean body mass index (BMI) was 23.9 kg/m^2^. As for cancer stage, 21 (10.1%), 29 (13.9%), 94 (45.2%), and 64 (30.8%) patients were diagnosed with stage 0, I, II, and III breast cancer, respectively. In addition, 113 patients (54.3%) underwent mastectomy, and, on average, 11.7 lymph nodes were dissected. Of the 184 patients (88.5%) who underwent chemotherapy, 169 were administered a taxane-based regimen. Of the 169 patients (81.3%) who underwent radiotherapy, 111 underwent direct radiotherapy to the regional lymph nodes. Due to unavailable data on radiotherapy performed at a different institution, it was difficult to determine whether direct radiotherapy to regional lymphatics was performed in five patients. The mean period from surgery to the diagnosis of BCRL was 291.2 days. As objective tests for diagnosis of BCRL, BIA was performed in 196 (94.2%) patients, and lymphoscintigraphy was performed in 95 (45.7%) patients.

### 3.2. Patients Dependent on Compression Management

Out of 208 patients, 125 (60.1%) were dependent on compression management despite 2 years of conservative care and were classified into the compression-dependent group. Results of the comparison of clinical characteristics between the compression-dependent and compression-free groups are presented in Table 2. There was no significant difference in patient demographics of the two groups. There was also no significant difference in patient compliance with conservative care. For tumor-related characteristics, cancer and node stages were significantly higher in the compression-dependent group (*p* = 0.002 and 0.003, respectively). Compression-dependent patients also exhibited more metastatic lymph nodes (*p* = 0.031). For treatment-related variables, more mastectomies and extensive lymph node resections were performed in the compression-dependent group (*p* = 0.044 and <0.001, respectively). More compression-dependent patients received taxane-based chemotherapy and underwent direct radiotherapy to the lymph nodes (*p* = 0.002 for both). For compression-dependent patients, the period from surgery to lymphedema diagnosis was significantly longer and the initial grade of BCRL based on the PEC was more severe (*p* = 0.010 and 0.002, respectively). Although average BIA parameters of both groups were higher than established cut-offs, compression-dependent patients showed poorer BIA results, especially SFBIA ratio with statistical significance (*p* = 0.001). All patients with T4 stage cancer or a history of cellulitis were dependent on compression management

### 3.3. Predictors of Dependence on Compression Management

Using univariate logistic regression analysis (Table 3), the association of clinical variables with dependence on compression was assessed. Of the tumor-related characteristics, more metastatic lymph nodes (odds ratio (OR), 1.096; 95% confidence interval (CI), 1.005–1.195), cancer stage III (OR, 4.000; 95% CI, 1.424–11.238), node stage N1 (OR, 2.527; 95% CI, 1.310–4.872) and N2 (OR, 4.263; 95% CI, 1.733–10.487) were significantly associated with dependence on compression management. Mastectomy (OR, 1.776; 95% CI, 1.014–3.112), five or more lymph node resections (OR, 3.831; 95% CI, 1.789–8.200), taxane-based chemotherapy (OR, 2.983; 95% CI, 1.454–6.119), and direct radiotherapy to the lymph nodes (OR, 2.471; 95% CI, 1.391–4.389) were statistically significant treatment-related factors. Of the swelling-related characteristics, dependence on compression was significantly associated with delayed-onset BCRL occurring at more than 1 year after surgery (OR, 2.961; 95% CI, 1.282–6.834) and with initially moderate to severe lymphedema, meaning PEC values above 10 (OR, 6.375; 95% CI, 1.432–28.378). Tumor stage T4 and cellulitis history could not be included in the logistic regression analysis because all these patients were compression-dependent.

Three predictors in the final model derived from multivariate analysis (Table 4) using forward stepwise variable selection were significantly associated with compression dependence after adjusting for other factors. When backward stepwise selection was applied, the same predictors remained significant. The dependence was significantly higher in patients with five or more lymph node resections (OR, 2.554; 95% CI, 1.121–5.820), those with direct radiotherapy to the lymph nodes (OR, 2.141; 95% CI, 1.138–4.029), and those with delayed-onset BCRL occurring more than 1 year after surgery (OR, 2.646; 95% CI, 1.058–6.614). Although not statistically significant, initially moderate to severe BCRL (PEC values over 10) was associated with compression dependence (OR, 4.430; 95% CI, 0.938–20.923).

### 3.4. Incidence and Risk of Compression-Dependent Patients According to the Number of Predictors

Table 5 showed that 23, 65, 95, and 20 patients had 0, 1, 2, and 3 predictors identified as significant, respectively. After 2 years of follow-up, 8 patients (34.8%) with no predictors, 28 (43.1%) with 1 predictor, 68 (71.6%) with 2 predictors, and 17 (85.0%) with 3 predictors required regular compression management. Using univariate analysis, those with 2 or 3 predictors showed a significantly increased risk of being compression-dependent by 4.722 or 10.825 times, respectively, compared to patients with no predictors.

## 4. Discussion

To our knowledge, this is the first study to approach persistent or progressive lymphedema based on whether interstitial fluid accumulation is ongoing, assessed in terms of compression dependence. The assessment was designed based on the assumption that patients with ongoing accumulation depend on compression despite efforts to taper it. Additionally, this was a retrospective cohort study in which patients with breast cancer received follow-up on lymphedema with surveillance and early management. The long-term effects of early intervention in BCRL have been unclear, but the results of this study may provide some clues. [4,5,30].

Predictors of patients with BCRL who required regular compression for at least 2 years were investigated in this study. Of the 208 patients, 125 (60.1%) were classified as compression-dependent. The significant predictors were resection of five or more lymph nodes, direct radiotherapy to the lymph nodes, and BCRL occurring more than 1 year after surgery. Initially moderate to severe lymphedema also showed substantial association with compression dependence. Patients with two or three predictors showed significantly increased dependence compared to those with no predictors.

So far, there have been many studies on the risk factors for BCRL [1,3,4,5,31,32,33,34]. The identified risk factors include obesity, mastectomy, extensive lymph nodes dissection, taxane-based chemotherapy, and radiotherapy to regional lymphatics. In comparison, relatively fewer reports investigated risk factors for persistent [6,7,8] or progressive [9,10,11] lymphedema. Initially severe lymphedema [6,7,10], lymph node metastases [7,9], radiotherapy to the lymph nodes [6,9,11], taxane-based chemotherapy [6], and weight gain [7] or obesity [9] were associated with the risk of persistent or progressive lymphedema. However, as described earlier, these previous studies simply defined persistent or progressive lymphedema based on increased limb volume. Chronic lymphedema results in fat deposition and fibrosis in soft tissue, which leads to irreversibly increased volume. Therefore, increased limb volume does not reflect the actual progress of interstitial fluid accumulation.

The present study overcame this limitation of previous reports. Three significant predictors were included in the final model derived from multivariate analysis. One of the three factors, direct radiotherapy to the axillary or supraclavicular lymph nodes, had been suggested as a major risk factor for persistent or progressive BCRL in previous studies [6,9,10]. The odds ratio had been reported as 2.74 [6] and 2.13 [9], respectively. This value was consistent with the value of 2.141 reported in our study. Another predictor, extensive lymph node dissection, had also been reported to be risk factor for BCRL [3,4,7,8,31,32]. This is an intuitive finding; however, no previous study has shown an association between lymph node dissection and persistent or progressive lymphedema [6,7,9,10,11]. Extensive lymph node dissection was defined as resection of five or more lymph nodes, as set by a previously published study [31]. The other factor identified in this study was delayed-onset BCRL. We defined delayed onset as longer than 1 year from surgery to diagnosis of lymphedema. This definition was based on previous research reporting that swelling in the first year after surgery tends to be transient [5]. This finding was also supported by other published studies [1,2].

Although not significant (*p* = 0.06), initially moderate to severe lymphedema (PEC values over 10) also increases compression dependence by four times. The association of initial severity with persistent or progressive lymphedema was supported by several studies [6,7,10]. Average PEC was 5.7 and 3.8 for compression-dependent and compression-free group, respectively. Although this value might suggest initial mild swelling, the duration of at least 3 months and conduct of BIA and lymphoscintigraphy enabled us to confirm the diagnosis of lymphedema. Thus, this study included many patients with initially mild BCRL, and investigated the time course of lymphedema, providing early active conservative care. It was also noteworthy finding that 2 of 19 (10.5%) patients with initially moderate to severe BCRL (PEC values over 10) could control their BCRL status without compression management; however, 68 of 144 (47.2%) patients with initially mild BCRL (PEC values below 5) regularly required compression management despite 2-year conservative care.

Although the association could not be assessed since all patients were classified into compression-dependent group, a history of cellulitis also seemed to be associated with compression dependence. Cellulitis is one of common complications in patients with lymphedema and known to cause the vicious cycle [35]. Specifically, cellulitis itself can impair the lymphatic system, and lead to lymphedema [36,37]. The underlying mechanism of lymphatic impairment in cellulitis was recently investigated: methicillin-resistant *S. aureus* exotoxins may cause death of lymphatic muscle cells which are critical for lymphatic contraction [37]. Accordingly, a history of cellulitis would have been a significant predictor of compression dependence, and this association should be explored in further studies.

As a secondary outcome, 125 of the 208 (60.1%) patients with BCRL were compression-dependent. The proportion is similar to that obtained in previous studies addressing persistent [6,7,8] or progressive [9,10,11] lymphedema.

This study allows the early prediction of patients with BCRL who will experience ongoing interstitial fluid accumulation despite early, active conservative care and should be considered for surgical treatment. Currently, physiologic procedures, such as LVA and VLNT, are considered the primary surgical options. However, patient selection and surgery timing pose as dilemmas in performing physiologic surgery. Conservative care may be sufficient for about 40–60% of BCRL patients with transient course. Meanwhile, physiologic surgery may be considered in patients with persistent or progressive BCRL. However, the surgery will be less effective after confirming BCRL progression because more pathologic changes will have occurred in the remaining lymphatics to be utilized for surgery. There have been a few guidelines regarding this dilemma [14,15,16,18,20,21,38]. In the guidelines, indocyanine green (ICG) lymphography was suggested as a potential assessment instrument for residual lymphatic function; however, the current ICG test protocol does not adequately reflect lymphatic transport and deep lymphatics [14,15,18,38,39]. Therefore, our current study may contribute to earlier patient selection and performance of the procedure, leading to decreased unnecessary surgery and enhanced effectiveness of physiologic surgery.

There are several limitations of this study. First, this study may have selection bias related to the study protocol only including patients who received 2 years of follow-up. Patients with long-term follow-up may have needed more intensive care. Second, the single-center study design limited the number of patients and study period. Although previous studies used a 2-year follow-up period, BCRL was also reported to have the potential for late progression more than 2 years after surgery in other studies [2,9,10,11]. A multi-centered study design would make it possible to enroll more patients and have a longer follow-up period. Third, the diagnosis of BCRL and the assessment of lymphedema status were made based on the circumference. The volume of both arms could not be obtained due to the retrospective nature and outpatient setting of the study. Finally, some clinical information, such as preoperative edema, was unavailable due to the study’s retrospective design. Previous studies mentioned that the prevalence of preoperative lymphedema was very low, and, therefore, not expected to be a major issue [3,6].

## 5. Conclusions

In conclusion, direct radiotherapy to regional lymphatics, resection of five or more lymph nodes, and delayed-onset BCRL occurring at least 1 year after surgery are significant predictors of patients with BCRL who will be dependent on compression management despite 2 years of conservative care. Initially moderate to severe BCRL and a history of cellulitis also seem to be strongly associated with compression-dependence. Our results allow for the early prediction of BCRL patients who should be considered for surgical treatment.

## Figures and Tables

**Table 1:** Baseline characteristics of the study population (*n* = 208).

Characteristics	Mean ± SD or *n* (%)
Patient demographics	
Age (Year)	49.0 ± 9.1
BMI (kg/m^2^)	23.9 ± 3.0
Tumor-Related	
Invasive Carcinoma	200 (96.2)
Number of Metastatic Lymph Nodes	2.8 ± 4.3
Cancer Stage	
0	21 (10.1)
I	29 (13.9)
II	94 (45.2)
III	64 (30.8)
Tumor Stage	
T0, Tis	24 (11.5)
T1	80 (38.5)
T2	76 (36.5)
T3	23 (11.1)
T4	5 (2.4)
Node Stage	
N0	64 (30.8)
N1	91 (43.8)
N2	37 (17.8)
N3	16 (7.7)
Treatment-Related	
Mastectomy	113 (54.3)
Number of Resected Lymph Nodes	11.7 ± 7.2
Chemotherapy	
All Regimen	184 (88.5)
Taxane-Based Regimen	169 (81.3)
Radiotherapy	
All Protocols	169 (81.3)
Direct to the Lymph Nodes, *n* = 203 ^a^	111 (53.4)
Swelling-Related	
Days from Surgery yo BCRL Diagnosis	291.2 ± 573.6
PEC	4.9 ± 4.7
Underwent BIA	196 (94.2)
Underwent Lymphoscintigraphy	95 (45.7)

SD, standard deviation; BMI, body mass index; BCRL, breast cancer-related lymphedema; PEC, percentage of excess circumference; BIA, bioimpedance analysis. ^a^ Data for 5 patients were unknown or not reported.

**Table 2:** Comparison of characteristics between the compression-dependent and compression-free groups.

Characteristics	Compression-Dependent (*n* = 125)	Compression-Free (*n* = 83)	*p*-Value
Patient Demographics			
Age (Year)	48.7 ± 9.4	49.5 ± 8.6	0.562
BMI (kg/m^2^)	24.0 ± 3.2	23.7 ± 2.6	0.379
Marital Status			
Married	103	72	
Not Married	22	11	0.401
Education, *n* = 207 ^a^			
MSC or Lower	17	12	
HSC or Further	107	71	0.879
Affected Side			
Right/Left	61/64	35/48	0.347
Dominant/Non-Dominant, *n* = 137 ^b^	40/38	34/25	0.461
Tumor-Related			
Invasive Carcinoma	123	77	0.061
Number of Metastatic Lymph Nodes	3.3 ± 4.6	2.0 ± 3.6	0.031
Cancer Stage			
0	9	12	
I	11	18	
II	57	37	
III	48	16	0.002
Tumor Stage			
T0, Tis	11	13	
T1	43	37	
T2	51	25	
T3	15	8	
T4	5	0	0.077
Node stage			
N0	27	37	
N1	59	32	
N2	28	9	
N3	11	5	0.003
Treatment-Related			
Mastectomy	75	38	0.044
Number of Resected Lymph Nodes	13.2 ± 6.9	9.5 ± 7.2	<0.001
Chemotherapy			
All Regimens	115	69	0.050
Taxane-Based Regimen	110	59	0.002
Radiotherapy			
All Protocols	103	66	0.602
Direct to the Lymph Nodes, *n* = 203 ^c^	77	34	0.002
Swelling-Related			
CDT start in Summer	29	20	0.881
Days from Surgery to BCRL Diagnosis	362.4 ± 709.1	184.0 ± 225.4	0.010
History of Cellulitis	10	0	0.007
Compliance to Conservative Care			
Good or Fair	111	73	
Poor	14	10	0.851
PEC	5.7 ± 5.3	3.8 ± 3.2	0.002
<5%	68	56	
5%–10%	40	25	
>10%	17	2	0.016
BIA Results			
ECF Ratio, *n* = 196 ^d^	1.0155 ± 0.0167	1.0108 ± 0.0164	0.055
SFBIA Ratio at 5 kHz, *n* = 196 ^d^	1.1195 ± 0.1701	1.0499 ± 0.0871	0.001
ECW/TBW, *n* = 196 ^d^	0.3897 ± 0.0085	0.3905 ± 0.0086	0.542

BMI, body mass index; MSC, middle school certificate; HSC, high school certificate; CDT, complex decongestive therapy; BCRL, breast cancer-related lymphedema; PEC, percentage of excess circumference; BIA, bioimpedance analysis; ECF, extracellular fluid; SFBIA, single frequency bioimpedance; ECW, extracellular water volume; TBW, total body water volume. ^a^ Data for 1 patient were unknown or not reported. ^b^ Data for 71 patients were unknown or not reported. ^c^ Data for 5 patients were unknown or not reported. ^d^ BIA was performed in 196 patients.

**Table 3:** Univariate logistic regression analysis for compression-dependent group (*n* = 208).

Variables	OR	95% CI	*p*-Value
Patient Demographics			
Age (Year)	0.991	(0.961, 1.022)	0.560
BMI (kg/m^2^)	1.041	(0.948, 1.144)	0.398
Not Married	1.398	(0.638, 3.062)	0.402
MSC or Lower, *n* = 207 ^a^	0.940	(0.423, 2.087)	0.879
Left Side Affected	0.765	(0.437, 1.338)	0.348
Dominant Side Affected, *n* = 137 ^b^	1.292	(0.654, 2.553)	0.461
Tumor-Related			
Invasive Carcinoma	4.792	(0.943, 24.348)	0.059
Number of Metastatic Lymph Nodes	1.096	(1.005, 1.195)	0.038
Cancer Stage (Reference: Stage 0)			
I	0.815	(0.259, 2.559)	0.726
II	2.054	(0.788, 5.355)	0.141
III	4.000	(1.424, 11.238)	0.009
Tumor Stage (Reference: T0, Tis)			
T1	1.373	(0.550, 3.340)	0.497
T2	2.411	(0.947, 6.139)	0.065
T3	2.216	(0.684, 7.177)	0.184
Node Stage (Reference: N0)			
N1	2.527	(1.310, 4.872)	0.006
N2	4.263	(1.733, 10.487)	0.002
N3	3.015	(0.938, 9.692)	0.064
Treatment-Related			
Mastectomy	1.776	(1.014, 3.112)	0.045
Number of Resected Lymph Nodes ≥5	3.831	(1.789, 8.200)	0.001
Chemotherapy	2.333	(0.983, 5.540)	0.055
Taxane-Based Chemotherapy	2.983	(1.454, 6.119)	0.003
Radiotherapy	1.206	(0.596, 2.439)	0.602
Direct Radiotherapy to The Lymph Nodes, *n* = 203 ^c^	2.471	(1.391, 4.389)	0.002
Swelling-Related			
CDT Start in Summer	0.952	(0.496, 1.827)	0.881
Days from Surgery to BCRL Diagnosis ≥1 year	2.961	(1.282, 6.834)	0.011
Poor Compliance	0.921	(0.388, 2.184)	0.851
PEC ≥ 10	6.375	(1.432, 28.378)	0.015

OR, odds ratio; CI, confidence interval; BMI, body mass index; MSC, middle school certificate; CDT, complex decongestive therapy; BCRL, breast cancer-related lymphedema; PEC, percentage of excess circumference. ^a^ Data for 1 patient were unknown or not reported. ^b^ Data for 71 patients were unknown or not reported. ^c^ Data for 5 patients were unknown or not reported.

**Table 4:** Multivariate logistic regression analysis for the compression-dependent group (*n* = 203).

Variables	OR	95% CI	*p*-Value
PEC ≥ 10	4.430	(0.938, 20.923)	0.060
Days from Surgery to BCRL Diagnosis ≥1 Year	2.646	(1.058, 6.614)	0.037
Number of Resected Lymph Nodes ≥5	2.554	(1.121, 5.820)	0.026
Direct Radiotherapy to Lymph Nodes	2.141	(1.138, 4.029)	0.018

OR, odds ratio; CI, confidence interval; PEC, percentage of excess circumference; BCRL, breast cancer-related lymphedema.

**Table 5:** Incidence and risk of the compression-dependent group according to the number of predictors.

No. of Predictors.	No. of Patients (*n* = 203)	Compression-Dependent (*n* = 121)	Compression-Free (*n* = 82)	OR	95% CI	*p*-Value
0	23	8 (34.8)	15 (65.2)	1.000		<0.001
1	65	28 (43.1)	37 (56.9)	1.419	(0.528, 3.813)	0.488
2	95	68 (71.6)	27 (28.4)	4.722	(1.796, 12.419)	0.002
3	20	17 (85.0)	3 (15.0)	10.825	(2.377, 47.503)	0.002

OR, odds ratio; CI, confidence interval.

## References

[1] Hayes S.C., Janda M., Cornish B., Battistutta D., Newman B. (2008). Lymphedema after breast cancer: Incidence, risk factors, and effect on upper body function. J. Clin. Oncol..

[2] Norman S.A., Localio A.R., Potashnik S.L., Simoes Torpey H.A., Kallan M.J., Weber A.L., Miller L.T., Demichele A., Solin L.J. (2009). Lymphedema in breast cancer survivors: Incidence, degree, time course, treatment, and symptoms. J. Clin. Oncol..

[3] DiSipio T., Rye S., Newman B., Hayes S. (2013). Incidence of unilateral arm lymphoedema after breast cancer: A systematic review and meta-analysis. Lancet Oncol..

[4] Rockson S.G. (2018). Lymphedema after breast cancer treatment. N. Engl. J. Med..

[5] Kilbreath S.L., Lee M.J., Refshauge K.M., Beith J.M., Ward L.C., Simpson J.M., Black D. (2013). Transient swelling versus lymphoedema in the first year following surgery for breast cancer. Support Care Cancer.

[6] Kim M., Shin K.H., Jung S.Y., Lee S., Kang H.S., Lee E.S., Chung S.H., Kim Y.J., Kim T.H., Cho K.H. (2016). Identification of prognostic risk factors for transient and persistent lymphedema after multimodal treatment for breast cancer. Cancer Res. Treat..

[7] Penn I.W., Chang Y.C., Chuang E., Chen C.M., Chung C.F., Kuo C.Y., Chuang T.Y. (2019). Risk factors and prediction model for persistent breast-cancer-related lymphedema: A 5-year cohort study. Support Care Cancer.

[8] Hayes S.C., Janda M., Ward L.C., Reul-Hirche H., Steele M.L., Carter J., Quinn M., Cornish B., Obermair A. (2017). Lymphedema following gynecological cancer: Results from a prospective, longitudinal cohort study on prevalence, incidence and risk factors. Gynecol. Oncol..

[9] Bar Ad V., Cheville A., Solin L.J., Dutta P., Both S., Harris E.E. (2010). Time course of mild arm lymphedema after breast conservation treatment for early-stage breast cancer. Int. J. Radiat. Oncol. Biol. Phys..

[10] Johansson K., Branje E. (2010). Arm lymphoedema in a cohort of breast cancer survivors 10 years after diagnosis. Acta Oncol..

[11] Bar Ad V., Dutta P.R., Solin L.J., Hwang W.T., Tan K.S., Both S., Cheville A., Harris E.E. (2012). Time-course of arm lymphedema and potential risk factors for progression of lymphedema after breast conservation treatment for early stage breast cancer. Breast J..

[12] Kim L., Jeon J.Y., Sung I.Y., Jeong S.Y., Do J.H., Kim H.J. (2011). Prediction of treatment outcome with bioimpedance measurements in breast cancer related lymphedema patients. Ann. Rehabil. Med..

[13] Do J.H., Choi K.H., Ahn J.S., Jeon J.Y. (2017). Effects of a complex rehabilitation program on edema status, physical function, and quality of life in lower-limb lymphedema after gynecological cancer surgery. Gynecol. Oncol..

[14] Brahma B., Yamamoto T. (2019). Breast cancer treatment-related lymphedema (BCRL): An overview of the literature and updates in microsurgery reconstructions. Eur. J. Surg. Oncol..

[15] Schaverien M.V., Coroneos C.J. (2019). Surgical treatment of lymphedema. Plast. Reconstr. Surg..

[16] Ogunleye A.A., Nguyen D.H., Lee G.K. (2020). Surgical treatment of lymphedema. JAMA Surg..

[17] Mihara M., Hara H., Hayashi Y., Narushima M., Yamamoto T., Todokoro T., Iida T., Sawamoto N., Araki J., Kikuchi K. (2012). Pathological steps of cancer-related lymphedema: Histological changes in the collecting lymphatic vessels after lymphadenectomy. PLoS ONE.

[18] Allen R.J., Cheng M.H. (2016). Lymphedema surgery: Patient selection and an overview of surgical techniques. J. Surg. Oncol..

[19] Bowman C., Piedalue K.A., Baydoun M., Carlson L.E. (2020). The quality of life and psychosocial implications of cancer-related lower-extremity lymphedema: A systematic review of the literature. J. Clin. Med..

[20] Markkula S.P., Leung N., Allen V.B., Furniss D. (2019). Surgical interventions for the prevention or treatment of lymphoedema after breast cancer treatment. Cochrane Database Syst. Rev..

[21] Ngo Q.D., Munot S., Mackie H., Czerniec S., Koelmeyer L.A., Lam T., Heydon-White A., Suami H., Boyages J. (2020). Vascularized lymph node transfer for patients with breast cancer-related lymphedema can potentially reduce the burden of ongoing conservative management. Lymphat. Res. Biol..

[22] Phillips G.S.A., Gore S., Ramsden A., Furniss D. (2019). Lymphaticovenular anastomosis improves quality of life and limb volume in patients with secondary lymphedema after breast cancer treatment. Breast J..

[23] Wolfs J., de Joode L., van der Hulst R., Qiu S.S. (2020). Correlation between patency and clinical improvement after lymphaticovenous anastomosis (LVA) in breast cancer-related lymphedema: 12-month follow-up. Breast Cancer Res. Treat..

[24] Suami H., Koelmeyer L., Mackie H., Boyages J. (2018). Patterns of lymphatic drainage after axillary node dissection impact arm lymphoedema severity: A review of animal and clinical imaging studies. Surg. Oncol..

[25] Liao S.F., Li S.H., Huang H.Y., Chen S.T., Kuo S.J., Chen D.R., Wei T.S. (2013). The efficacy of complex decongestive physiotherapy (CDP) and predictive factors of lymphedema severity and response to CDP in breast cancer-related lymphedema (BCRL). Breast.

[26] Erdogan Iyigun Z., Selamoglu D., Alco G., Pilanci K.N., Ordu C., Agacayak F., Elbuken F., Bozdogan A., Ilgun S., Guler Uysal F. (2015). Bioelectrical impedance for detecting and monitoring lymphedema in patients with breast cancer. Preliminary results of the florence nightingale breast study group. Lymphat. Res. Biol..

[27] Jung M., Jeon J.Y., Yun G.J., Yang S., Kwon S., Seo Y.J. (2018). Reference values of bioelectrical impedance analysis for detecting breast cancer-related lymphedema. Medicine.

[28] Qin E.S., Bowen M.J., James S.L., Chen W.F. (2020). Multi-segment bioimpedance can assess patients with bilateral lymphedema. J. Plast. Reconstr. Aesthet. Surg..

[29] International Lymphoedema Framework. Best Practice for the Management of Lymphedema. https://www.lympho.org/portfolio/best-practice-for-the-management-of-lymphoedema/.

[30] Specht M.C., Miller C.L., Russell T.A., Horick N., Skolny M.N., O’Toole J.A., Jammallo L.S., Niemierko A., Sadek B.T., Shenouda M.N. (2013). Defining a threshold for intervention in breast cancer-related lymphedema: What level of arm volume increase predicts progression?. Breast Cancer Res. Treat..

[31] Kilbreath S.L., Refshauge K.M., Beith J.M., Ward L.C., Ung O.A., Dylke E.S., French J.R., Yee J., Koelmeyer L., Gaitatzis K. (2016). Risk factors for lymphoedema in women with breast cancer: A large prospective cohort. Breast.

[32] Li X., Huang H., Lin Q., Yu Q., Zhou Y., Long W., Wang N. (2017). Validation of a breast cancer nomogram to predict lymphedema in a Chinese population. J. Surg. Res..

[33] Armer J.M., Ballman K.V., McCall L., Ostby P.L., Zagar E., Kuerer H.M., Hunt K.K., Boughey J.C. (2019). Factors associated with lymphedema in women with node-positive breast cancer treated with neoadjuvant chemotherapy and axillary dissection. JAMA Surg..

[34] Byun H.K., Chang J.S., Im S.H., Kirova Y.M., Arsene-Henry A., Choi S.H., Cho Y.U., Park H.S., Kim J.Y., Suh C.O. (2019). Risk of lymphedema following contemporary treatment for breast cancer: An analysis of 7617 consecutive patients from a multidisciplinary perspective. Ann. Surg..

[35] Park S.I., Yang E.J., Kim D.K., Jeong H.J., Kim G.C., Sim Y.J. (2016). Prevalence and Epidemiological Factors Involved in Cellulitis in Korean Patients with Lymphedema. Ann. Rehabil. Med..

[36] de Godoy J.M., de Godoy M.F., Valente A., Camacho E.L., Paiva E.V. (2000). Lymphoscintigraphic evaluation in patients after erysipelas. Lymphology.

[37] Jones D., Meijer E.F.J., Blatter C., Liao S., Pereira E.R., Bouta E.M., Jung K., Chin S.M., Huang P., Munn L.L. (2018). Methicillin-resistant Staphylococcus aureus causes sustained collecting lymphatic vessel dysfunction. Sci. Transl. Med..

[38] Narushima M., Yamamoto T., Ogata F., Yoshimatsu H., Mihara M., Koshima I. (2016). Indocyanine green lymphography findings in limb lymphedema. J. Reconstr. Microsurg..

[39] Wiser I., Mehrara B.J., Coriddi M., Kenworthy E., Cavalli M., Encarnacion E., Dayan J.H. (2020). Preoperative assessment of upper extremity secondary lymphedema. Cancers.

